# Caveolin-1 Scaffolding Domain Peptides Alleviate Liver Fibrosis by Inhibiting TGF-β1/Smad Signaling in Mice

**DOI:** 10.3390/ijms19061729

**Published:** 2018-06-11

**Authors:** Jing Lu, Jie Zhang, Yan Wang, Quan Sun

**Affiliations:** Department of Laboratory Animal Science, School of Basic Medical Science, Capital Medical University, Beijing 100069, China; lujing@ccmu.edu.cn (J.L.); 13811560655@163.com (J.Z.); Sugar06240713@163.com (Y.W.)

**Keywords:** liver fibrosis, caveolin-1, hepatic stellate cells, TGF-β1/Smad signaling

## Abstract

Liver fibrosis is the common pathological process characterized by activation of hepatic stellate cells (HSCs) and overproduction of extracellular matrix (ECM). Caveolin-1 (Cav1), the principal component of caveolae, is regarded as an important inhibitor of multiple signaling molecules including transforming growth factor β1(TGF-β1) signaling. To evaluate the role of Cav1 in liver fibrosis, Cav1 deficient (Cav1^−/−^) and wild type (WT) mice were subjected to liver fibrosis induced by carbon tetrachloride (CCl_4_). Results indicated no significant difference between Cav1^−/−^ and WT mice in inflammation or collagen content before CCl_4_ treatment. After CCl_4_ administration, Cav1^−/−^ mice showed enhanced TGF-β1 signaling, as reflected by a significantly greater amount of phosphorylation of Smad2 and collagen deposition in livers over WT animals. Qualitative and quantitative analysis indicated that inflammatory injury to the liver was markedly aggravated, accompanied by increased degeneration and necrosis of hepatocytes, higher alanine aminotransferase (ALT)/aspartate aminotransferase (AST), TGF-α and IL-1β levels in Cav1^−/−^ animals. The mRNA and protein levels of α-smooth muscle actin (α-SMA), Collagen α1(I), and Collagen α1(III) were further enhanced in Cav1^−/−^ animals. We also observed a significant decrease in collagen content in Cav1^−/−^ and WT animals administrated with Cav1 scaffolding domain peptides (CSD). In vitro study indicated that phosphorylation of Smad2 was inhibited after CSD treatment, accompanied by decreased protein levels of α-SMA, Collagen α1(I), and Collagen α1(III) in HSCs. We conclude that Cav1 is an important inhibitor of TGF-β1/Smad signaling in HSCs activation and collagen production, which might make it a promising target for therapy of liver fibrosis.

## 1. Introduction

Liver fibrosis is a common pathological process underlying most types of chronic liver disease. It is characterized by overproduction of extracellular matrix (ECM). Advanced liver fibrosis leads to cirrhosis, liver failure, and portal hypertension and often requires liver transplantation. Many patients present initially in the clinic with advanced fibrosis or cirrhosis, which are largely irreversible. In this way, antifibrotics that prevent progression of fibrosis toward cirrhosis or induce reversal of advanced fibrosis are urgently needed [1,2,3].

Caveolin-1 (Cav1), the principal coat protein of caveolae, might act as a therapeutic target for treatment of liver fibrosis [4]. Caveolae were initially found in the plasma membrane by electron microscopy and are involved in vesicular trafficking, endocytosis, and in the compartmentalization of specific signaling cascades [5]. There are three members in the caveolin family of caveolae coat proteins. Cav1 and Cav2 are mostly expressed in endothelial cells, adipocytes, and fibroblasts; Cav3 is muscle specific [6,7]. Besides being a structural protein involved in the formation and function of plasma membrane caveolae, Cav1 also serves as a regulator of multiple signaling molecules including the MAP kinase family, the protein kinase C family, G proteins, growth factor receptor tyrosine kinases, non-receptor tyrosine kinases (NTKs), Akt and eNOS activation, and TGFβ-induced signaling [8,9,10,11,12,13,14,15]. The interaction between Cav1 and kinases frequently inhibits their activity. It has been shown that Cav1 deficiency hyperactivates signaling molecules in vitro and in vivo [16,17,18].

Cav1 can bind to multiple kinases and thereby inhibit their activity. This ability has been mapped to a sequence known as the Cav1 scaffolding domain peptides (CSD, amino acids 82–101 of Cav1). When connected with antennapedia homeodomain (AP), CSD can quickly enter cells and tissues [19,20]. CSD is particularly helpful because it is functional when delivered in vivo [21,22]. It has been confirmed that intratracheal administration of the CSD can prevent the development of bleomycin-induced lung fibrosis in mice [23,24]. Furthermore, CSD protects against cryoablation-induced myocardial fibrosis by inhibition of TGF-β1 signaling [25,26]. However, it is still unknown whether CSD has potential beneficial effects of in liver fibrosis.

In this study, we hypothesized that Cav1 deficiency would enhance TGF-β1/Smad signaling and CSD could inhibit the progression of liver fibrosis by restoring the inhibitory effect of Cav1. A mouse model induced by carbon tetrachloride was used to identify the role of Cav1 in activation of hepatic stellate cells (HSCs) and collagen production in the progress of liver fibrosis. We also studied TGF-β1/Smad signaling in isolated HSCs from Cav1 deficiency and WT mice.

## 2. Results

### 2.1. Downregulation of Cav1 after CCl_4_ Injection

**Figure 1:**
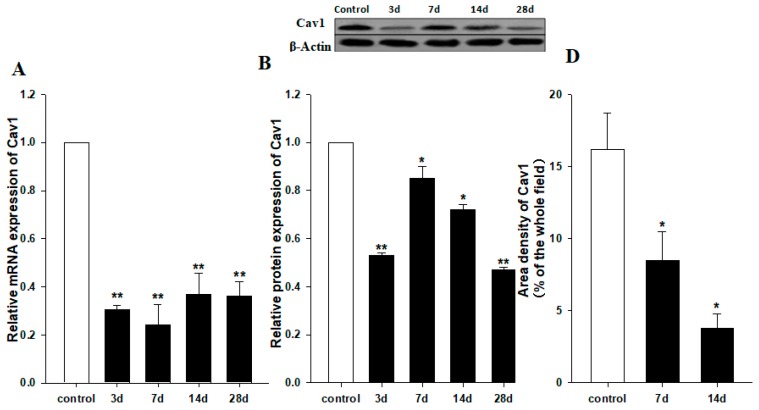

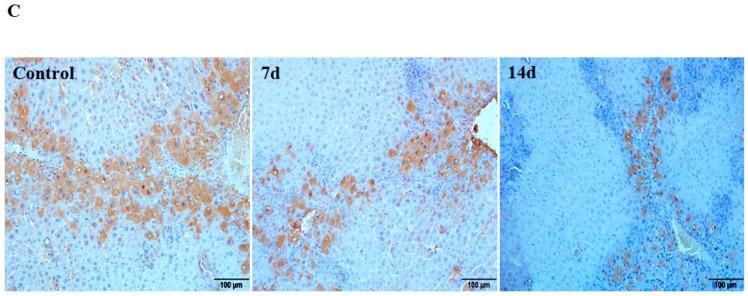
Reduced expression of Cav1 in livers of WT mice after CCl_4_ injection. (**A**) Cav1 mRNA was significantly lower in WT mice than control mice 3, 7, 14, and 28 days after CCl_4_ injection. Gene expression was assessed by qRT-PCR and normalized to β-actin. WT control group value has been used for normalization among study groups. (**B**) Measurement of Cav-1 protein expression in livers of control and 3, 17, 14, and 28 days post CCl_4_ injection. In insert, a representative Western blot from which these data were obtained. (**C**) Representative immunohistochemistry staining for Cav-1. (**D**) Area density of Cav1 staining in representative images for each group. This protein levels dramatically decreased at 7 (middle) and 14 days (right) after CCl_4_ injection. *n* = 10, bar represents mean ± SD, ** *p* < 0.01, * *p* < 0.05, compared with control animals, bar = 100 μm.

To study the role of Cav1 in liver fibrosis, we measured the expression level of Cav1 after CCl_4_ injection in liver tissues of WT animals by semi-quantitative real-time PCR and Western blot. As shown in Figure 1, the Cav1 mRNA levels were decreased in CCl_4_-treated mice compared to control animals after 3, 7, 14, and 28 days (** *p* < 0.01, Figure 1A). Western blot assays confirmed a decrease in Cav1 protein from day 3 to day 28 (** *p* < 0.01, * *p* < 0.05, Figure 1B). To identify the cell type responsible for reduced expression of Cav1, liver sections were performed by immunohistochemistry staining. The results indicated that Cav1 expressions in hepatic stellate cells or cholangiocytes (Figure 1C) were significantly reduced in CCl_4_-treated mice compared to control animals 7 and 14 days after CCl_4_ injection (* *p* < 0.05, Figure 1D).

### 2.2. Enhanced Inflammation Response in Cav1^−/−^ Mice

**Figure 2:**
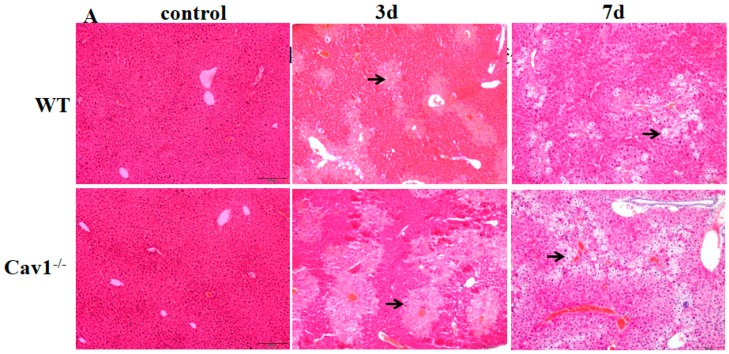

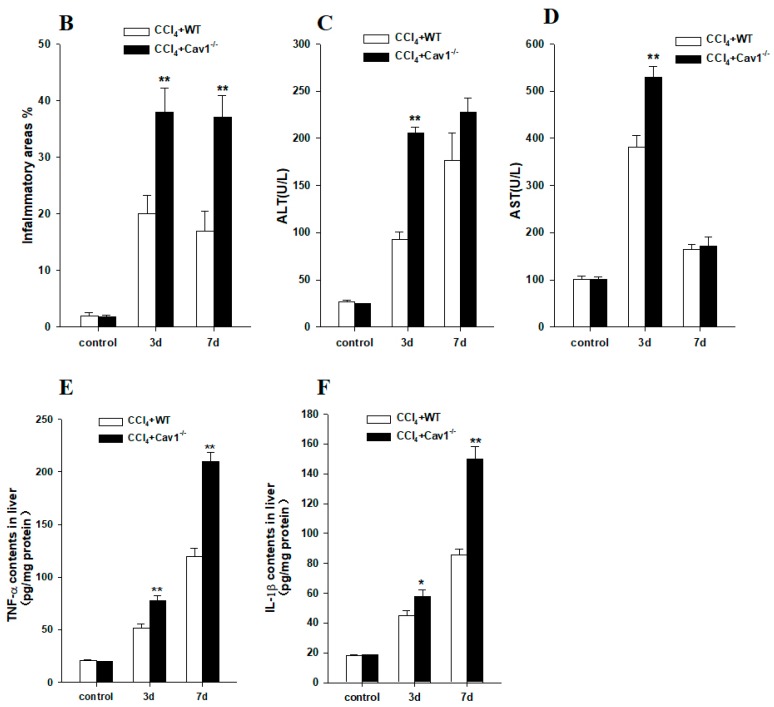
Increased inflammatory injury in Cav-1^−/−^ livers. (**A**) Representative photomicrographs of HE-stained liver sections from CCl_4_ treated WT and Cav1^−/−^ mice. Degenerated (middle) and necrotic (right) hepatocytes were observed at three and seven days after CCl_4_ injection, respectively. (**B**) The quantitative analysis of liver inflammation. Significantly increased inflammatory areas were present in Cav1^−/−^ livers compared to WT. ALT (**C**); and AST (**D**) levels were detected by BS-200 Chemistry Analyzer (MINDRAY, Shenzhen, China). TGF-α (**E**); and IL-1β (**F**) levels were measured by ELISA kits (R&D Systems, Shanghai, China). *n* = 10, bar represents mean ± SD, ** *p* < 0.01, * *p* < 0.05, compared with WT at the same time point, bar = 200 μm.

To investigate the role of Cav1 in CCl_4_ induced inflammation in livers, we measured the histopathological lesions of livers in Cav1^−/−^ and WT mice following CCl_4_ injection. There was no spontaneous inflammation in the control Cav1^−/−^ livers. Degeneration and necrosis of hepatocytes were observed in Cav1^−/−^ and WT mice after three and seven days (Figure 2A). There were significantly more areas of degeneration and necrosis in Cav1^−/−^ mice than in WT (** *p* < 0.01, Figure 2B). Similarly, the ALT and AST levels in Cav1^−/−^ mice were also significantly higher than that in WT animals three days after CCl_4_ treatment (** *p* <0.01, Figure 2C,D). As shown in Figure 2E,F, pro-inflammatory cytokines of TGF-α and IL-1β were highly induced in Cav1^−/−^ compared to WT mice (** *p* <0.01, * *p* <0.05, Figure 2E,F). These data suggest that Cav1 deficiency aggravates inflammatory extent of CCl_4_-induced liver injury.

### 2.3. Increased Collagen Production in Cav1^−/−^ Mice

**Figure 3:**
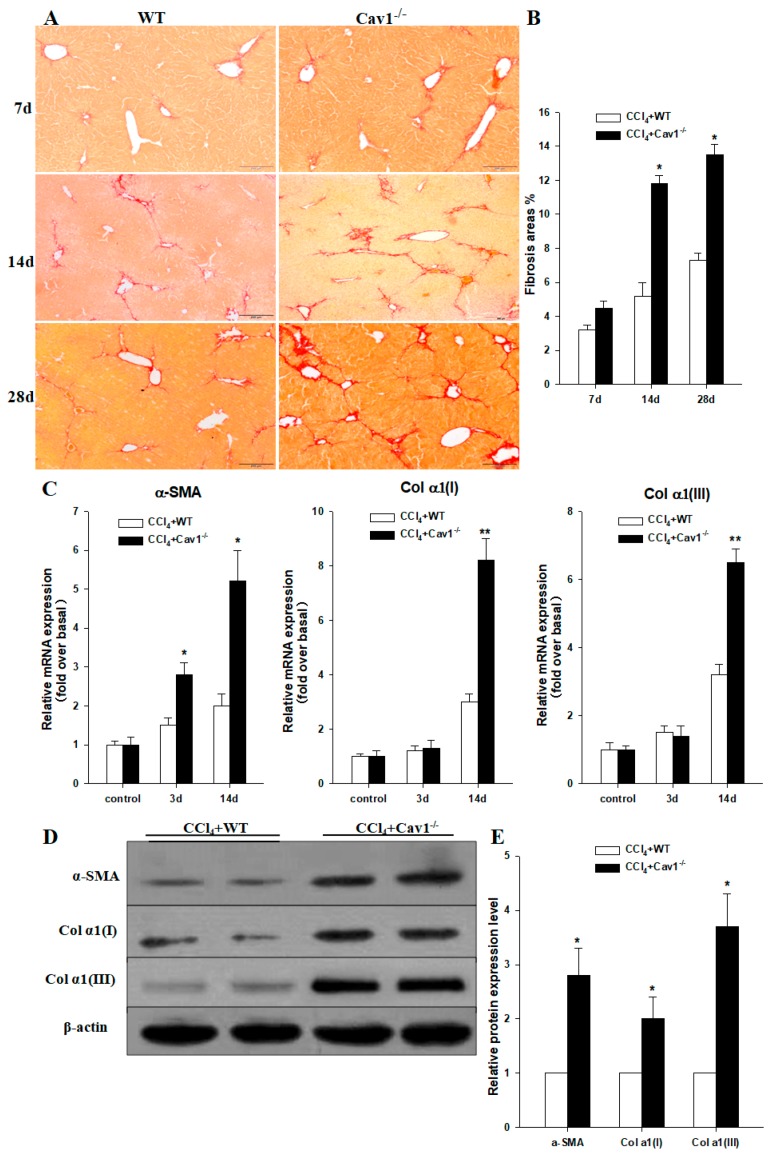
Increased collagen deposition in CCl_4_ treated Cav1^−/−^ mice. (**A**) Representative photomicrographs of Sirius red stained liver sections from CCl_4_ treated WT and Cav-1^−/−^ mice. The collagen network is stained in red. The extent of collagen deposition was increased from day 7 to day 28. (**B**) Quantitative analysis demonstrated that Cav1^−/−^ mice had significantly more collagen deposition than WT. (**C**) The mRNA expression of fibrotic markers were quantified using real-time RT-PCR. 18s rRNA was used for normalization of PCR data for the genes of α-SMA, Col α1(I) and Col α1(III). (**D**) Immunoblotting for α-SMA, Col α1(I), Col α1(III) in liver tissues of Cav1^−/−^ and WT animals at 14 days post CCl_4_ treatment. (**E**) Histogram showing densitometry analysis and quantification of α-SMA, Col α1(I), Col α1(III). The signals for α-SMA, Col α1(I), Col α1(III) were normalized to respective bands for β-actin. *n* = 10, bar represents mean ± SD, ** *p* < 0.01, * *p* < 0.05, compared with WT at the same time point, bar = 200 μm.

To evaluate the role of Cav1 in the progress of liver fibrosis, Cav1^−/−^ and WT animals were treated with CCl_4_ to induce liver fibrosis. After 7, 14, and 28 days, liver tissues were collected for histopathology examination. As shown in Figure 3A, the results of Sirius Red staining revealed that there was no difference between Cav1^−/−^ and WT animals in collagen deposition on Day 7. However, the collagen deposition in livers was significantly increased in Cav1^−/−^ compared with WT animals on Days 14 and 28 (Figure 3B, * *p* < 0.05).

To investigate the effect of Cav1 on HSCs activation, we measured the mRNA and protein levels of α-SMA, Col α1(I) and Col α1(III) in liver tissues of Cav1^−/−^ and WT animals by real-time qPCR and Western blotting after CCl_4_ administration. Results showed that there was no significant difference in α-SMA, Col α1(I) and Col α1(III) mRNA levels in control animals from Cav1^−/−^ and WT groups. At 3 and 14 days, the mRNA levels of α-SMA were increased significantly in Cav1^−/−^ mice compared to WT. Similarly, the mRNA levels of Col α1(I) and Col α1(III) were increased significantly in Cav1^−/−^ mice compared to WT 14 days after CCl_4_ treatment (Figure 3C, ** *p* < 0.01, * *p* < 0.05). The results of Western blot also showed significant increases of α-SMA, Col α1(I) and Col α1(III) in livers (Figure 3D) of Cav1^−/−^ mice 14 days post CCl_4_ injection compared to those of WT mice (Figure 3E, * *p* < 0.05).

### 2.4. Enhanced Activation of TGF-β1/Smad Signaling in Cav1^−/−^ Mice

**Figure 4:**
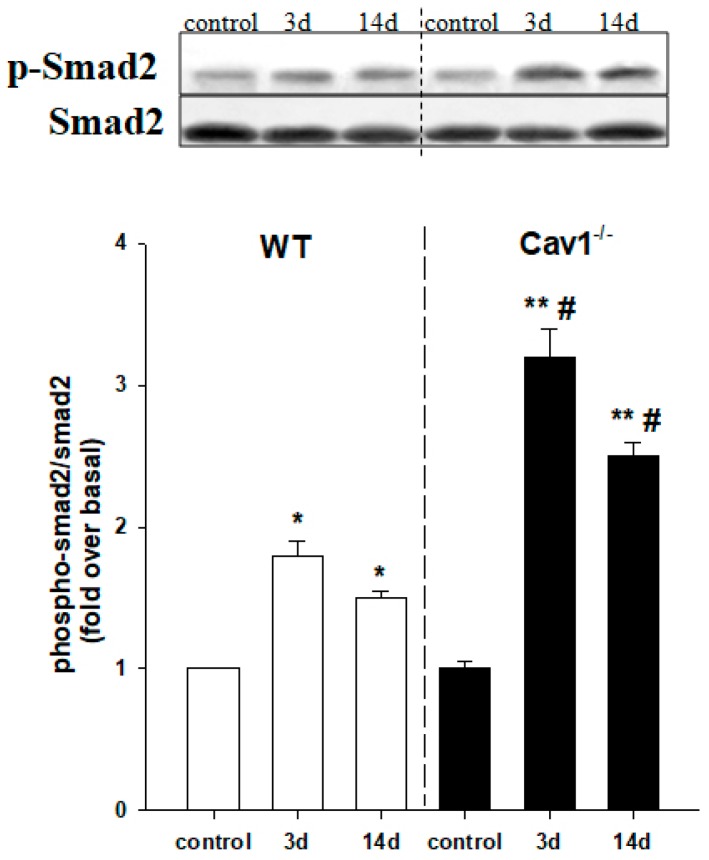
Enhanced activation of TGF-β signaling in Cav1^−/−^ miceActivation of TGF-β signaling was assessed by measuring the protein expression levels of p-Smad2 and total Smad2 in mouse livers following CCl_4_ treatment. The increased ratio of p-Smad2 to Smad2 indicates the activation of TGF-β1/Smad signaling. Enhanced activation of TGF-β signaling was present in Cav1^−/−^ mice at 3 and 14 days post CCl4 treatment. *n* = 10, bar represents mean ± SD, ** *p* < 0.01, * *p* < 0.05, compared with control for the same strain; ^#^ *p* < 0.05 compared with WT for the same time point.

To evaluate whether Cav1 regulates activation of TGF-β1/Smad signaling, we measured the protein expression levels of p-Smad2 and total Smad2 in livers of both Cav1^−/−^ and WT mice by Western blot. The results indicated that p-Smad2 expression levels were increased and total Smad2 levels remained the same in livers after CCl_4_ treatment for 3 and 14 days in WT mice. Interestingly, this increase was enhanced in the Cav1^−/−^ livers compared to WT (Figure 4, ** *p* < 0.01, * *p* < 0.05 compared to control in the same strain; ^#^ *p* < 0.05 compared to WT). The increased ratio of p-Smad2 to Smad2 indicates the activation of TGF-β1/Smad signaling. Therefore, these results indicate there is an inverse correlation between the expression of Cav1 and the activation of TGF-β1/Smad signaling in the progress of liver fibrosis.

### 2.5. Enhanced Activation of HSCs in Cav1^−/−^ Mice

**Figure 5:**
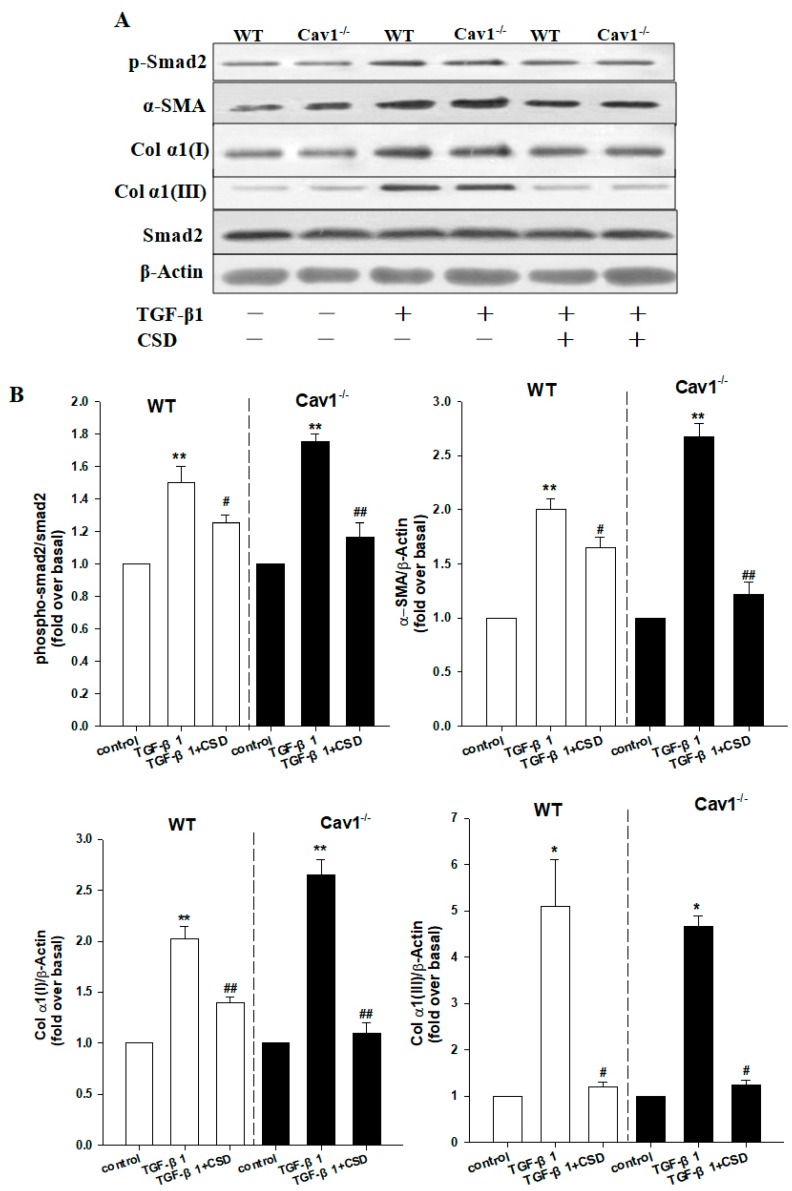
CSD inhibits activation of HSCs. (**A**) Immunoblotting for p-Smad2, α-SMA, Col α1(I) and Col α1(III) following stimulation with TGF-β1 (5 ng/mL) for 24 h in the presence or absence of Cav1 scaffolding domain peptide (CSD, 10 mM). (**B**) Histogram showing densitometry analysis and quantification of p-Smad2, α-SMA, Col α1(I), and Col α1(III). The signals for α-SMA, Col α1(I), Col α1(III) and Smad2 were normalized to respective bands for β-actin and/or Smad2. Bar represents mean ± SD. ** *p* < 0.01, * *p* < 0.05, compared to control for the same strain; ^##^ *p* < 0.01, ^#^ *p* < 0.05 compared with TGF-β1 group for the same strain, *n* = 3.

**Figure 6:**
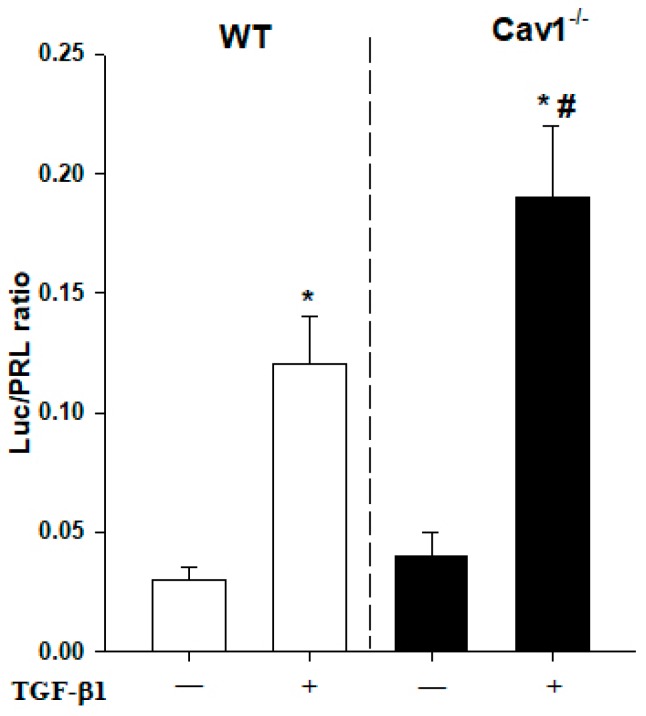
Enhanced activity of TGF-β1 signaling in Cav1^−/−^ HSCs Primary mouse HSCs extracted from WT and Cav1^−/−^ mice were cotransfected with SMAD luciferase reporter plasmid and pRL-TK vector. Twenty-four hours after transfection, cells were treated with TGF-β1 (5 ng/mL) for another 24 h. Luciferase activity was determined with the commercial luciferase reporter assay system. Each value represents the mean ± SD of at least three independent transfection experiments, each performed in triplicate. * *p* < 0.05 compared with no TGF-β1 group for the same strain; ^#^ *p* < 0.05 compared with TGF-β1-treated HSCs in WT mice.

To confirm Cav1 negatively regulated the activation of TGF-β1/Smad signaling, we studied the response of isolated HSCs of Cav1^−/−^ and WT mice to TGF-β1 stimulation. We assessed the protein expression levels of p-Smad2, α-SMA, Col α1(I) and Col α1(III) and found that levels of these proteins were significantly increased after TGF-β1 treatment for 24 h (Figure 5, ** *p* < 0.01, * *p* < 0.05 vs control), which were more pronounced in the Cav1^−/−^ HSCs compared to WT. The increased protein levels of p-Smad2, α-SMA, Col α1(I), and Col α1(III) became significantly lower after the CSD treatment (Figure 5, ^##^ *p* < 0.01, ^#^ *p* < 0.05 vs TGF-β1). We also analyzed the expression level of luciferase gene reporter and found that the activity of transfected reporter constructs was more significant in the Cav1^−/−^ HSCs (Figure 6, ^#^ *p* < 0.05 vs WT HSCs). These results confirmed that TGF-β1/Smad signaling is negatively regulated by Cav1 in HSCs activation.

### 2.6. CSD Alleviates CCl_4_-Induced Liver Fibrosis

**Figure 7:**
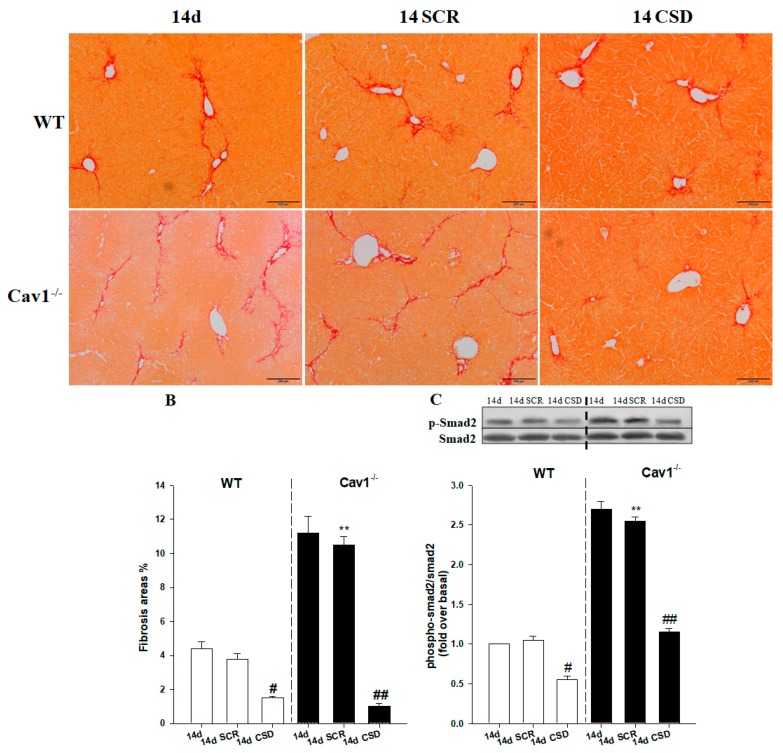
CSD alleviates liver fibrosis by inhibition of Smad2 phosphorylation. (**A**) Representative photomicrographs of Sirius red stained livers from CCl_4_-treated WT and Cav1^−/−^ mice. The collagen network is stained in red. The scar contained a large amount of collagen at 14 days post injury when the mice were treated with the scrambled peptide (SCR). There is less collagen deposition in the livers of mice treated with the CSD peptide for both strains. (**B**) Qualitative analysis of Sirius red stained livers demonstrated that Cav1^−/−^ mice had significantly more collagen deposition than WT animals at 14 days when treated with SCR. Administration of the CSD peptide prevented the accumulation of collagen. (**C**) The p-Smad2 expression following CCl_4_ injection treated with CSD was reduced in both Cav1^−/−^ and WT mice. *n* = 10, bar represents mean ± SD. ** *p* < 0.01 compared with 14-day SCR group in WT mice; ^##^ *p* < 0.01, ^#^ *p* < 0.05 compared with 14-day SCR group for the same strain, bar = 200 μm.

To identify Cav1 as an important negative regulator in the process of liver fibrosis, we delivered CSD to WT and Cav1^−/−^ animals following CCl_4_ treatment and assessed whether CSD could inhibit the progress of fibrosis. The results of Sirius Red staining revealed that collagen production was significantly reduced in both WT and Cav1^−/−^ mice treated with CSD, compared to mice that were treated with SCR (** *p* < 0.01 vs. 14-day SCR in WT; ^##^ *p* < 0.01 and ^#^ *p* < 0.05 vs. 14-day SCR in the same strain, Figure 7A,B). There was no significant difference in collagen deposition between the SCR and control group. More interestingly, the decrease of collagen deposition was more enhanced in the Cav1^−/−^ mice after treatment with CSD compared to WT. Furthermore, we also found that animals treated with the CSD had decreased ratio of p-Smad2 to Smad2 (** *p* < 0.01 vs. 14-day SCR in WT; ^##^ *p* < 0.01 and ^#^ *p* < 0.05 vs. 14-day SCR in the same strain, Figure 7C). These data indicated that Cav1 negatively regulated the activation of TGF-β1/Smad signaling.

## 3. Discussion

Previous studies have confirmed Cav1 as a promising anti-fibrotic target in various types of fibrotic diseases including lung, cardiac, and kidney fibrosis by both animal and pharmacological models. Although the interaction between Cav1 and multiple signaling molecules such as MAP kinase family and Smad has been studied extensively, it is still not clear whether these Cav1-mediated signaling pathways can be targeted as prospective therapies in liver fibrosis [4,9,25,26,27]. 

In this study, we found mRNA and protein levels of Cav1 were significantly reduced in the progress of liver fibrosis. Deficiency of Cav1 led to increased collagen deposition and enhanced activation of TGF-β1/Smad signaling. Furthermore, we also found restoration of Cav1 with CSD treatment could reduce collagen production and phosphorylation of Smad2 in both Cav1 deficiency and WT mice, and interestingly the decrease in collagen deposition and phosphorylation of Smad2 were more enhanced in Cav1 deficiency mice. These data indicated Cav1 might act as a negative regulator in collagen production through inhibition of TGF-β1/Smad signaling. Similarly, a recent study also revealed that Cav1 deficiency aggravated CCl_4_ induced liver fibrosis in mice by regulation of oxidative stress [28]. Besides, it was reported that Cav1 might negatively regulate human liver fibrosis. Cav1 expression has been shown to be reduced and inversely correlated with high levels of miR-199a-5p, which is elevated in patients with liver fibrosis [29].

HSCs play a central role for excess collagen synthesis during liver fibrosis [30,31]. Collagen gene expression is regulated by TGF-β1 and Smad family activation in tissues [32,33].We found that Cav1 deficiency promoted activation of HSCs by up-regulating TGF-β1/Smad signaling. What is even more exciting is that CSD treatment reduces activation of HSCs and collagen release by down-regulating TGF-β1/Smad signaling. Therefore, Cav1 might block TGF-β1 signaling pathway through directly interacting with TGF-β receptor I and inhibiting the downstream phosphorylation of Smad2, as previously reported [12,34]. The mechanism by which Cav1 reduces TGF-β1/Smad signaling pathway in HSCs still requires further study. 

Growing evidence has linked inflammation to tissue damage and liver fibrosis in conditions such as drug-induced liver injury, alcoholic steatohepatitis, and nonalcoholic steatohepatitis [27,35,36,37]. We measured increased degeneration and necrosis of hepatocytes in Cav1^−/−^ and WT mice three and seven days post injury but the increase was more significant in the Cav1^−/−^ mice. Similarly, ALT/AST, TGF-α and IL-1β Levels in Cav1^−/−^ mice were higher than that in WT mice. A previous report showed that Cav1 is essential for protecting against binge drinking-induced liver damage by inhibiting reactive nitrogen species [37], and our findings also suggest that Cav1 plays an important role in reducing CCl_4_-induced liver injury. Similarly, Cav1 deletion exacerbates cardiac interstitial fibrosis by promoting M2 macrophage activation in mice after myocardial infarction. It has been confirmed the reason is that M2 macrophages, known for promoting scar formation, accumulate more in Cav1^−/−^ mice compared to WT mice. Further studies showed that the survival rate in Cav1^−/−^ mice that received macrophages from WT mice was increased and survival rates for WT mice which received Cav1^−/−^ macrophages were decreased [25].

## 4. Materials and Methods

### 4.1. Mouse Strain

Cav1 knock-out mice (Cav1^−/−^, STOCK Cav1^tm1Mls^/J) and WT B6129SF2/J controls were purchased from the Jackson Laboratory and were used for in vivo and in vitro experiments. All experimental design and animal treatments were conducted in accordance with the Guidelines of the Animal Experiments and Experimental Animals Management Committee of Capital Medical University. The study protocol was approved by the Animal Experiments and Experimental Animal Welfare Committee of Capital Medical University (Permit Number: AEEI-2016-150, 16, October, 2016).

### 4.2. Mouse Models and CSD Treatment

Male mice, 8–10 weeks old, weighing 25–28 g were included in this study (*n* = 10). A mouse model of liver fibrosis was induced by injection of CCl_4_. Mice received intraperitoneal injections of CCl_4_/olive oil (OO) mixture (1:9 *v*/*v*) at 1 μL/g body weight, twice per week. Control mice were administrated with OO at 1 μL/g body weight twice-weekly. Mice were sacrificed at 3, 7, 14, and 28 days after CCl_4_ treatment by CO_2_ exposure. The liver tissues were collected for immunohistochemical staining, Sirius Red staining, RT-PCR, and Western blotting. Serum was isolated for test of the serum ALT and AST level. Ten additional male Cav1^−/−^ and WT mice were daily treated intraperitoneally with the CSD (4 mg/kg) or its scrambled peptide (SCR, 4 mg/kg). Whole livers were collected 14 days post CCl_4_ treatment for histological examination and mRNA and protein expression detection.

CSD (amino acids 82-101 of Cav1; DGIWKASFTTFTVTKYWFYR) and SCR (WGIDKAFFTTSTVTYKWFRY) were synthesized and analyzed by mass spectrometry to confirm purity more than 97% by Sangon Biotech Co., Ltd. (Shanghai, China). Desiccated peptides were weighed, and dissolved in DMSO to 10 mM, and then diluted with distilled water to 1 mM.

### 4.3. Isolation and Culture of Primary Mouse HSCs

Primary mouse HSCs were isolated from adult male Cav1^−/−^ and WT mice by collagenase perfusion and purified by density gradient in Nicodenz (AXIS-SHIELD PoC, Dundee, Scotland). Isolated HSCs were incubated in Dulbecco’s modified Eagle’s medium (DMEM) supplemented with 10% fetal bovine serum (FBS). To investigate the effect of Cav1 on HSCs activation and collagen production, HSCs were treated with TGF-β1 (5 ng/mL, Abcam, Shanghai, China) in the presence or absence of 1 ml of serum-free DMEM containing 5 μM CSD for 24 h. After 6 h, the culture medium and cell layer were harvested. HSCs were serum starved for 24 h before TGF-β1 treatment.

### 4.4. Biochemical Measurements

On Days 3 and 7 after CCl_4_ treatment, serum was isolated by orbital venous plexus blood collection. Serum AST and ALT levels were detected by standard enzymatic assay kits. The highly-colored end product was measured at 490–520 nm by a spectrophotometer (Hitachi 736–10, Beijing, China). The absorbance of each end product is proportional to the enzyme’s activity. TNF-α and interleukin-1β (IL-1β) expression levels in livers were measured by ELISA kits (R&D Systems, Minneapolis, MN, USA) following the manufacturer’s instructions.

### 4.5. Assessment of Liver Fibrosis

In brief, liver tissues were collected and fixed in 4% paraformaldehyde for 24 h and then embedded in paraffin. Four micron-thick paraffin sections were deparaffinized and stained with Sirius Red solution for 1 h. The fibrotic area was measured by computer-assisted image analysis with Leica Qwin software (V3, Lecia Microsystems, Heidelberg, Germany). The mean value of 15 randomly selected areas for each sample was used to evaluate the percentage of fibrotic area.

### 4.6. qRT-PCR

Total RNA was extracted from liver tissue and analyzed by quantitative PCR as previously reported [25,26]. Primers were designed as follows: 18S rRNA: sense, 5′-GTA ACC CGT TGA ACC CCA TT-3′; antisense, 5′-CCA TCC AAT CGG TAG TAG CG-3′. Mouse a-SMA: sense, 5′-ATG CTC CCA GGG CTG TTT T-3′; antisense, 5′-TTC CAA CCA TTA CTC CCT GATGT-3′. Mouse Collagen α1(I): sense, 5′-AGG GCG AGT GCT GTG CTT T-3′; antisense, 5′-CCC TCG ACT CCT ACATCT TCT GA-3′. Mouse Collagen α1(III): sense, 5′-TGA AAC CCC AGC AAA ACA AAA-3′; antisense, 5′-TCA CTTGCA CTG GTT GAT AAG ATT AA-3′. Mouse Caveolin-1: sense, 5′ ACA GTT TCG ACG GCA TCT GG-3′; antisense, 5′-CAA AGA GTG GAT CGC AGA AG-3′.

### 4.7. Immunohistochemical Staining

Immunohistochemical staining for Cav1 (Cat. No.610406, 1:1000, BD Biosciences, Shanghai, China) was performed according to the manufacturer’s instructions by immunohistochemistry kits (Boster Biological Engineering Co., Wuhan, China). The yellow-stained areas in the sections were analyzed with an image analyzer (Image-Pro Plus, MediaCybernetics, Rockville, MD, USA) for semi-quantitative analysis. The results were shown as the area density (area of the positive cells/area of the whole field).

### 4.8. Western Blot

The expression levels of proteins in livers and mouse HSCs were analyzed as previously described [35]. Primary antibodies were as follows: anti-mouse Cav1 (Cat. No.610406, 1:1000, BD Biosciences), anti-mouse α-SMA (Cat. No.A5228, 1:1000, Sigma-Alorich; Shanghai, China), anti-mouse Collagen α1(I) (Cat. No.ab6308, 1:200, Abcam), anti-mouse Collagen α1(III) (Cat. No.ab7778, 1:200, Abcam), anti phospho-Smad2 (Cat. No.ab53100, 1:1000, Abcam), anti-Smad2 (Cat. No.ab33875, 1:1000, Abcam), anti-β-Actin (Cat. No.12262, 1:1000, Cell Signaling, Shanghai, China). Goat anti-mouse IgG labeled with HRP (Cat. No.ab6789, 1:2000, Abcam) was used as secondary antibodies. 

### 4.9. Reporter Gene Assays

Primary mouse HSCs received Lipofectamine 2000 (Life Technologies, Carlsbad, CA, USA) with TGF-β1/Smad responsive reporter genes REPOTMSMAD (Genomeditech Co., Shanghai, China) as previously described [36,37]. Transfection efficiency was normalized by cotransfection of Renilla luciferase reporter plasmid pRL-TK (Promega, Madison, WI, USA). Twenty-four hours after the transfection, HSCs were stimulated with TGF-β1 (5 ng/mL) for another 24 h. The results were obtained from three wells processed in parallel and normalized with Renilla luciferase activity.

### 4.10. Statistical Analysis

Data are presented as mean ± standard deviation (SD). The data between groups were analyzed for statistical differences using SPSS 17.0 statistical software (SPSS Institute, Chicago, IL, USA) and one-way analysis of variance tests plus subsequent Bonferroni post hoc test. The p-value was two-tailed and considered as statistically significant or very significant if it was less than 0.05 or 0.01, respectively.

## 5. Conclusions

In this study, we evaluated the expression of Cav1 and its role on the activation of TGF-β1/Smad signaling in vivo and in vitro. In the WT mice, we measured decreased expression of Cav1 during the early stage of liver fibrosis. By contrast, we found deficiency of Cav1 to be associated with more collagen deposition. Interestingly, restored expression of Cav1 could reduce collagen production by inhibition of TGF-β1/Smad signaling in vivo and in vitro. This study indicates that Cav1 has a pivotal role in activation of HSCs and collagen production and suggests a potential therapeutic target for liver fibrosis.
