# Correction: Lu, J. et al. Caveolin-1 Scaffolding Domain Peptides Alleviate Liver Fibrosis by Inhibiting TGF-β1/Smad Signaling in Mice. *Int. J. Mol. Sci.*, 2018, *19*, 1729

**DOI:** 10.3390/ijms20081866

**Published:** 2019-04-15

**Authors:** Jing Lu, Jie Zhang, Yan Wang, Quan Sun

**Affiliations:** Department of Laboratory Animal Science, School of Basic Medical Science, Capital Medical University, Beijing 100069, China; lujing@ccmu.edu.cn (J.L.); 13811560655@163.com (J.Z.); Sugar06240713@163.com (Y.W.)

The author wishes to make the following correction to this paper [1]. Due to mislabeling, Figure 3 should be replaced with the following:


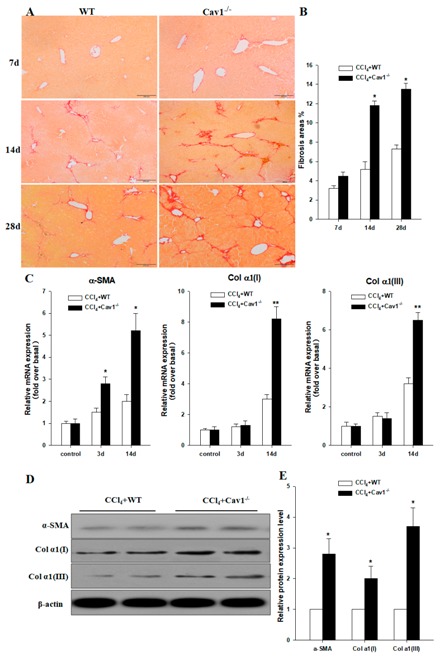


These changes have no material impact on the conclusions of our paper. The authors would like to apologize for any inconvenience caused to the readers by these changes.
